# Quercetin carbon quantum dots: dual-target therapy for intracerebral hemorrhage in mice

**DOI:** 10.1186/s13041-024-01159-6

**Published:** 2025-03-03

**Authors:** Guangyu Jia, Xinyu Yang, Yamei Yu, Yuanyuan Li, Zhe Zhang, Xiaolong Tang, Qi Wang, Heqing Zheng, Yao Xiao, Shiyong Li, Ye Wang

**Affiliations:** 1 Department of Neurology, The Second Affiliated Hospital, Jiangxi Medical College, Nanchang University, Nanchang, Jiangxi 330006 China; 2 Department of Neurosurgery, The Second Affiliated Hospital, Jiangxi Medical College, Nanchang University, Nanchang, Jiangxi 330006 China; 3 Institute of Neuroscience, Nanchang University, Nanchang, Jiangxi 330006 China; 4 Jiangxi Province Key Laboratory of Neurological Diseases, Nanchang, Jiangxi 330006 China; 5 JXHC Key Laboratory of Neurological Medicine, Nanchang, Jiangxi 330006 China

**Keywords:** Intracerebral hemorrhage, Quercetin, Carbon quantum dots, Iron overload, Oxidative stress

## Abstract

Following intracerebral hemorrhage, mitigating oxidative stress and removing excess iron are critical strategies for reducing secondary brain injury and improving neurological outcomes. In vitro, we synthesized quercetin-ethylenediamine carbon quantum dots (QECQDs) with diameters of 2–11 nm and found that QECQDs effectively scavenge ABTS+· and DPPH· free radicals, defending HT22 cells against hemin-induced oxidative stress. In vivo, QECQDs predominantly accumulate in the pia mater, subarachnoid space, and dura mater after intrathecal injection. Compared to the ICH injury group, QECQDs treatment effectively improves cerebral blood flow, inhibits oxidative stress damage, and reduces neuron death. Importantly, QECQDs treatment reduced hemorrhage volume, alleviated edema, and improved neurological function. This lays a foundation for developing multi-target drugs for treating ICH.

**Graphical Abstract:**

**Figure:**
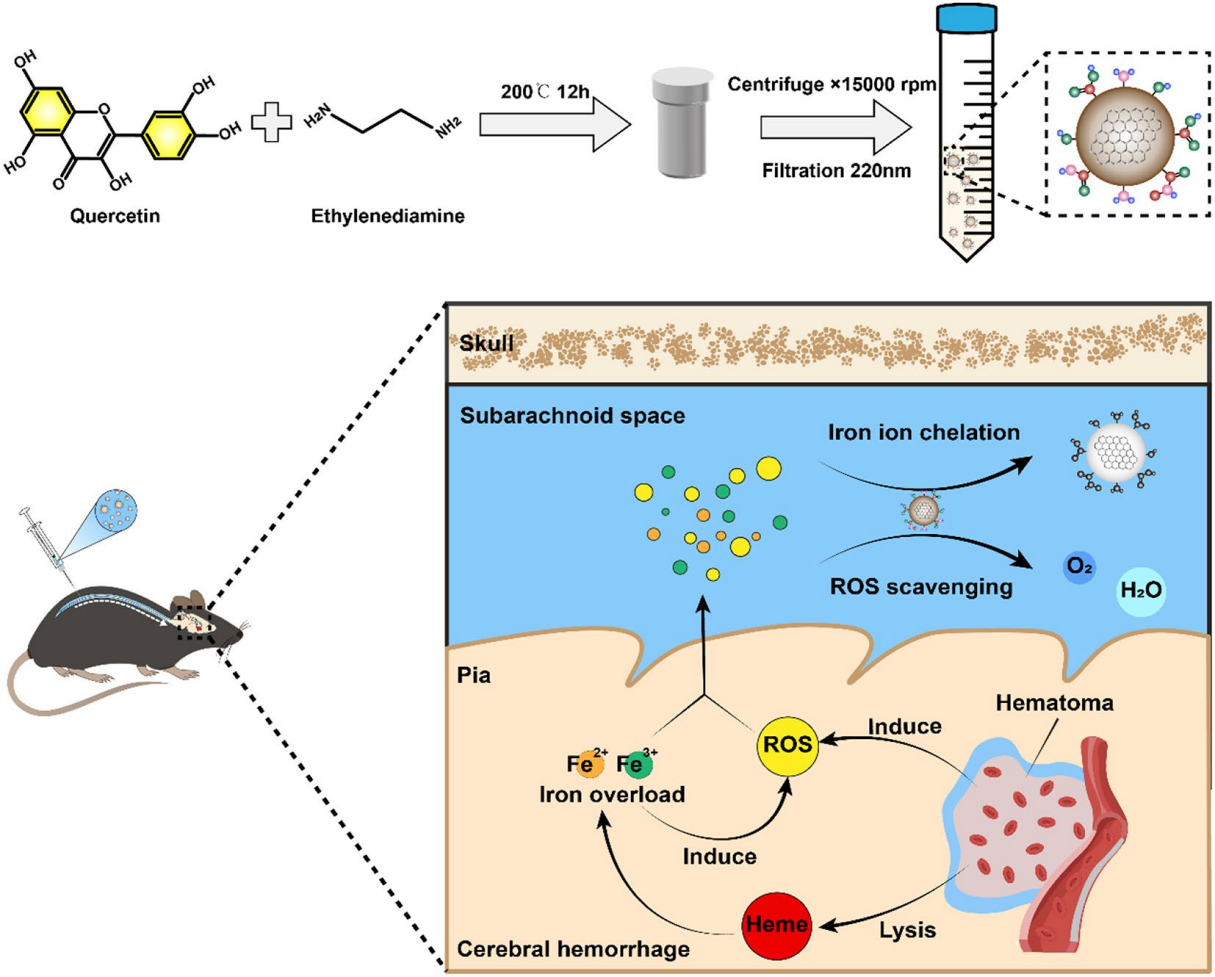
**TOC**. Synthesis of QECQDs and their application in treating intracerebral hemorrhage

**Supplementary Information:**

The online version contains supplementary material available at 10.1186/s13041-024-01159-6.

## Introduction

Intracerebral hemorrhage (ICH), or cerebral hemorrhage, results from a brain blood vessel rupture, causing bleeding into surrounding tissue. It is associated with high disability and mortality rates [1, 2]. Following ICH, erythrocyte hemolysis induces iron overload, leading to heightened ROS production and causing oxidative injury to cellular constituents like lipids, proteins, and DNA. This cascade activates apoptotic signaling pathways, culminating in aberrant neuronal and glial cell loss [3, 4]. ICH can induce neuronal damage and functional impairment through multiple mechanisms, contradicting current single-target therapeutic drugs. Therefore, developing multi-target drugs is crucial for effective treatment of ICH.

Quercetin (3,5,7,3’,4’-Pentahydroxyflavone.) is a natural compound with diverse biological activities, including antioxidant properties [5], anti-inflammatory effects [6–8], anticancer properties [9], cardiovascular protection [10–12], antiallergic effects [13], antiviral activity [14], neuroprotection [15–17], and antidiabetic effects [18–20]. Despite its multi-target therapeutic effects, quercetin’s poor water solubility and instability in vivo severely limit its application [21].

Quantum dots are inorganic nanomaterials with low biotoxicity, excellent biocompatibility, and remarkable optical properties, among others, playing a beneficial role in various models of neurological disorders. Our previous studies have indicated that intrathecal injection of ginsenoside Rb1 carbon quantum dots effectively scavenges excessive reactive oxygen species, treating hemorrhagic stroke in mice and improving neural function [22]. Furthermore, administration of aspirin carbon quantum dots in vivo demonstrates excellent meningeal anti-inflammatory and excess iron chelating effects in a mouse model of brain hemorrhage [23]. Nasal delivery of microfluidically synthesized ultra-small chitosan/graphene quantum dot particles (CS/GQD NPs) in the treatment of streptozotocin (STZ)-induced Alzheimer’s disease-like rat models significantly enhances memory [24]. In vitro experiments have shown that graphene carbon quantum dots and nitrogen-doped graphene carbon quantum dots promote neurite outgrowth in N2A cell lines and exhibit enhanced neurotrophic activity [25]. Therefore, this study aims to leverage the excellent biological properties of quercetin quantum dots and the advantages of multi-target therapy in treating ICH, advancing the development of quercetin drugs.

In this study, we synthesized quercetin carbon quantum dots and found in vitro experiments that they effectively chelate iron ions and scavenge ROS. Following intrathecal injection, QECQDs primarily distribute in the subarachnoid space and brain surface, effectively improving cerebral surface blood flow. They efficiently clear accumulated iron ions and oxygen radicals within the brain, improving the microenvironment on the injured side, protecting vulnerable neurons, and enhancing neurological function. This study may offer a new perspective on developing multi-target quercetin drugs for treating ICH.

## Results and discussion

### Nanoproperties of QECQDs

**Fig. 1:**
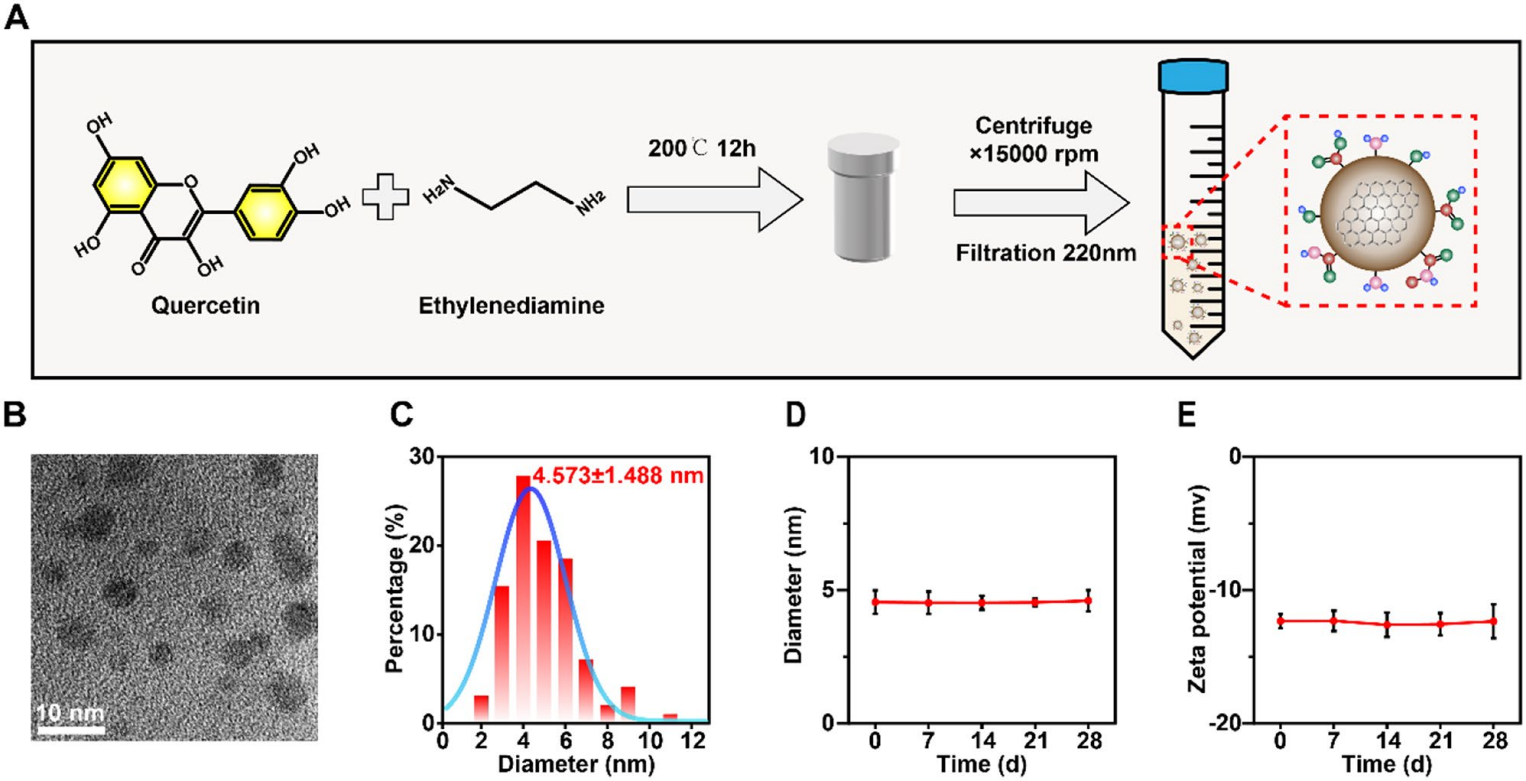
Preparation and Characterization of QECQDs. (**a**) Schematic illustration of the QECQDs synthesis process. (**b**) TEM image of QECQDs. Scale bar, 10 nm. (**c**) The hydrodynamic diameter of QECQDs. Particle size (**d**) and ζ-potential (**e**) of QECQDs were monitored over 0–28 days

As shown in Fig. 1a, QECQDs were synthesized using quercetin and ethylenediamine as precursors via hydrothermal synthesis at 200 degrees Celsius. Transmission electron microscopy (TEM) images revealed uniformly dispersed spherical QECQDs with diameters ranging from 2 to 11 nm (Fig. 1b). Dynamic light scattering (DLS) analysis revealed the mean hydrodynamic diameter of QECQDs to be 4.573 ± 1.488 nm. Interestingly, this is consistent with the particle size of QECQDs observed in TEM. (Fig. 1c). Additionally, we assessed the stability of QECQDs over an extended period (Fig. 1d-e), demonstrating good stability in terms of particle size and zeta potential over 28 days. This observation demonstrates our proficient synthesis of quercetin-derived carbon quantum dots, characterised by their remarkable stability.

### Optical properties of QECQDs

**Fig. 2:**
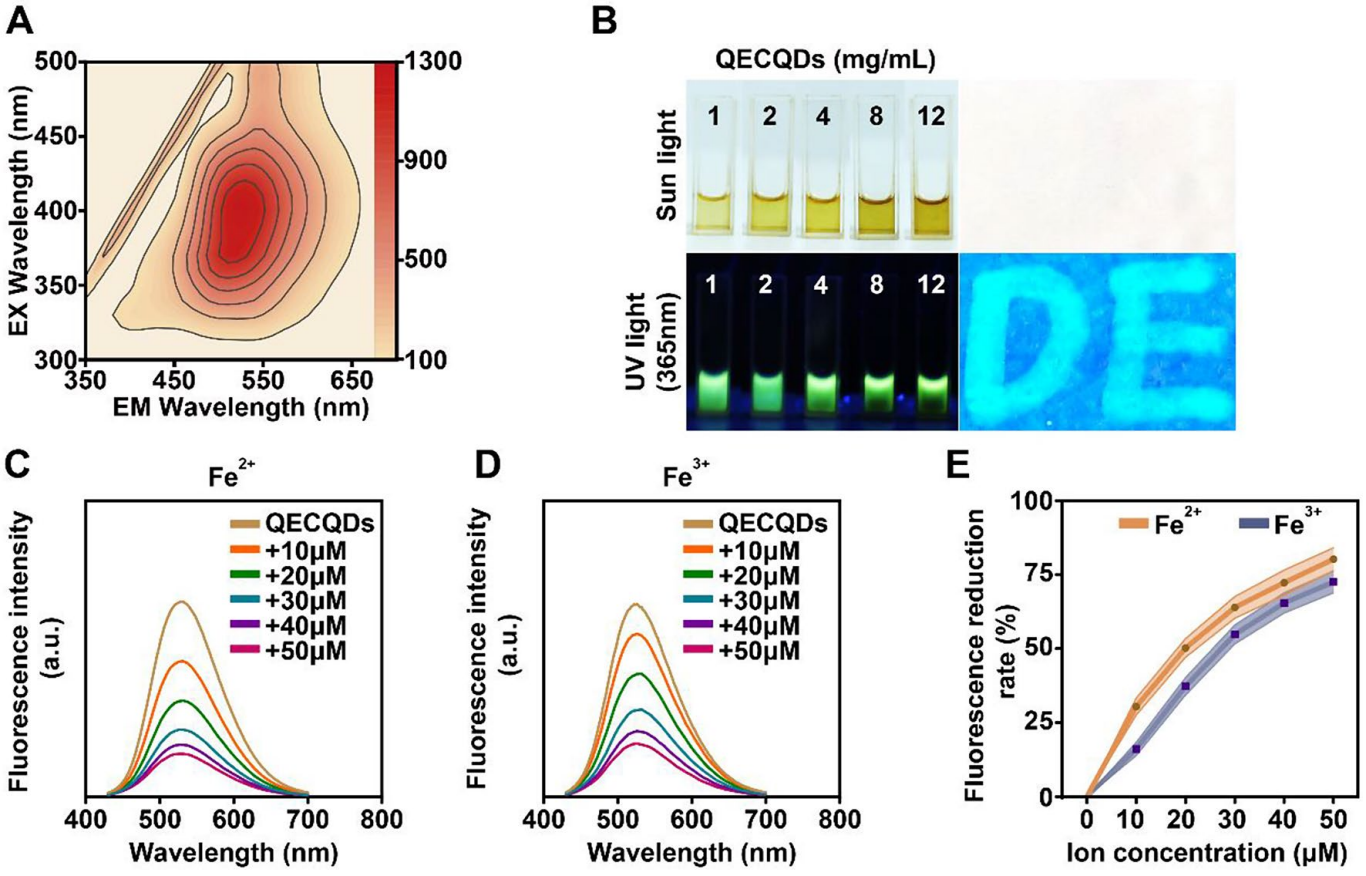
Fluorescence properties and iron ion chelation performance of QECQDs. (**a**) Three-dimensional fluorescence spectrum of QECQDs showing optimal excitation (λ_ex_ = 400 nm) and emission wavelengths (λ_em_ = 525 nm). (**b**) Images of QECQDs solutions at various concentrations (1, 2, 4, 8, 12 mg/mL) under natural and 365 nm UV light demonstrate the fluorescence anti-counterfeiting feature of 1 mg/mL QECQDs. (**c**, **d**) Fluorescence intensity curves of QECQDs incubated with different concentrations of Fe^2+^ (**c**) and Fe^3+^ (**d**) ions (0, 10, 20, 30, 40, and 50 µM). (**e**) The fluorescence intensity rate decreased QECQDs incubated with different concentrations of Fe^2+^ and Fe^3+^ ions (0, 10, 20, 30, 40, and 50 µM) (*n* = 5/group, one-way ANOVA)

As shown in Fig. 2a, The three-dimensional fluorescence emission spectrum of QECQDs showed that the optimal excitation wavelength was 400 nm and the corresponding emission wavelength was 525 nm. The emission spectrum peaks predominantly between 500 and 550 nm, showing pronounced excitation-dependent characteristics with variations in both wavelength and intensity of emission. This variability may be attributed to quantum dot size effects and the diversity of surface emissive sites in QECQDs [26]. As shown in Fig. 2b, solutions of QECQDs at different concentrations appear yellow-brown under natural sunlight and cyan-green under UV light at 365 nm. Subsequently, we investigated the anti-counterfeiting properties of QECQDs by writing the letters “DE” using a 1 mg/mL quantum dot solution. Under 365 nm UV light, the letters emitted intense cyan-green fluorescence. This suggests that the quercetin-derived carbon quantum dots synthesized in our study manifest favorable optical properties, thereby highlighting their potential utility in biomedical research and clinical applications.

### QECQDs chelate iron ions

To investigate the iron chelation properties of QECQDs, we co-incubated solutions of ferrous sulphate and ferric sulphate at different concentrations (0, 10, 20, 30, 40, and 50 µM) with QECQDs dissolved in artificial cerebrospinal fluid (Fig. 2c, d) at a concentration of 1 mg/mL.

Experimental data indicated that under a 400 nm excitation wavelength, the fluorescence intensity of QECQD solutions gradually decreased with increasing concentrations of ferrous and ferric ions. Quantitative analysis (Fig. 2e) showed fluorescence quenching efficiency exceeded 70% when ion concentrations reached 50 µM. These data demonstrate the strong ability of QECQDs to chelate Fe^2+^ and Fe^3+^ ions. In summary, these findings establish a robust foundation for effectively utilizing QECQDs as potent iron chelators in vivo, offering promising prospects for mitigating iron-induced toxicity.

### QECQDs scavenge free radicals

Quercetin (Que) is widely sourced and possesses potent antioxidant capabilities as a natural scavenger of free radicals [27–29]. However, its poor water solubility limits its biocompatibility and restricts its application in disease treatment [30, 31]. We synthesized QECQDs with improved solubility and evaluated the scavenging abilities of Que and QECQDs against two free radical reagents, ABTS+· and DPPH·. In solution, ABTS+· and DPPH· demonstrate characteristic blue and purple colors, with absorption peaks at 734 nm and 515 nm, respectively, enabling quantification of antioxidant capacity through a reduction in absorbance upon reaction with antioxidant substances [32].

**Fig. 3:**
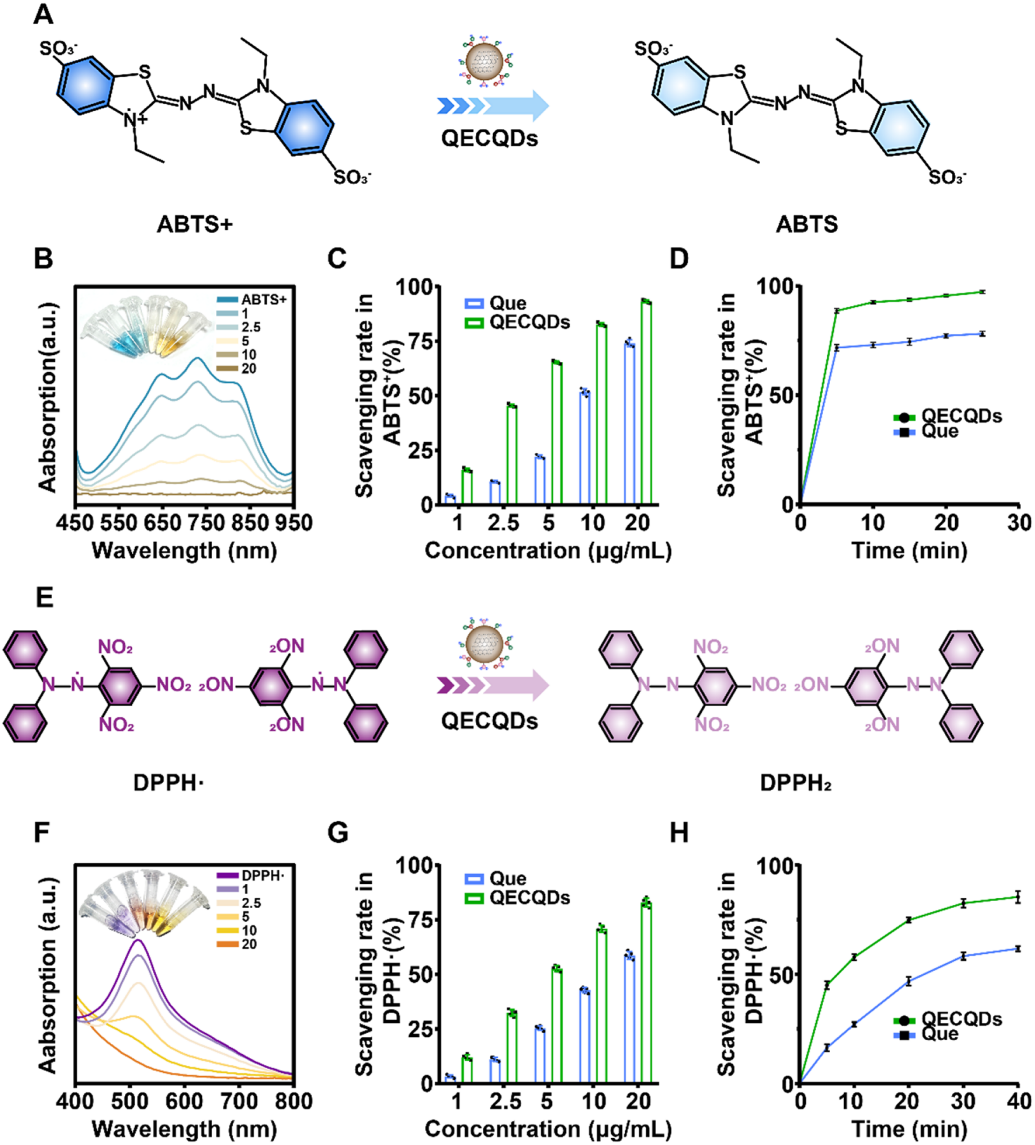
Free radical scavenging ability of QECQDs. (**a**) Schematic diagram of the scavenging ability of QECQDs on ABTS+·. (**b**) UV-visible spectra of ABTS + exposed to different concentrations of QECQDs (0, 1, 2.5, 5, 10, and 20 µg/mL). Inset: Photograph showing the color of the reaction solution. (**c**) ABTS+·scavenging ability of QECQDs and Quercetin at different concentrations (0, 1, 2.5, 5, 10 and 20 µg/mL) (*n* = 5/group, one-way ANOVA). (**d**) Time-clearance kinetic curve study of ABTS+·clearance ability in the presence of QECQDs and Quercetin (*n* = 5/group, one-way ANOVA). (**e**) Schematic diagram of the scavenging ability of QECQDs for DPPH·. (**f**) UV-visible spectra of DPPH. Exposed to different concentrations of QECQDs (0, 1, 2.5, 5, 10, and 20 µg/mL). Inset: Photograph showing the color of the reaction solution. (**g**) DPPH·scavenging capacity of different concentrations of QECQDs and Quercetin (0, 1, 2.5, 5, 10 and 20 µg/mL) (*n* = 5/group, one-way ANOVA). (**h**) Time-clearance kinetic curve study of DPPH·clearance capacity in the presence of QECQDs and Quercetin (*n* = 5/group, one-way ANOVA)

Initially, we assessed the free radical scavenging capability of QECQDs using the ABTS+· standard assay reagent in PBS (Fig. 3a). At various concentrations (0, 1, 2.5, 5, 10, and 20 µg/mL) of QECQDs, the solution color changed from blue to colorless as QECQDs concentration increased (Fig. 3b). Quantitative results (Fig. 3c) demonstrated a concentration-dependent clearance of ABTS+· radicals by QECQDs, with a clearance rate of 93.60% observed at 20 µg/mL after 15 min of incubation. Subsequently, we investigated the time-dependent scavenging of ABTS+· radicals by QECQDs (20 µg/mL) incubated at room temperature for different durations (0, 5, 10, 15, 20, and 25 min), as shown in the quantitative results (Fig. 3d). Furthermore, we evaluated the ability of QECQDs to scavenge DPPH· radicals (Fig. 3e). As QECQDs concentration increased (0, 1, 2.5, 5, 10, and 20 µg/mL), the UV absorption peak of DPPH· at 515 nm decreased gradually, changing the solution color from purple to colorless and finally yellow (Fig. 3f). Quantitative experimental results (Fig. 3g) indicated a concentration-dependent clearance of DPPH· radicals by QECQDs, with an 82.52% clearance rate observed at 20 µg/mL after 30 min of incubation. Subsequently, we examined the time-dependent scavenging of DPPH· radicals by QECQDs (20 µg/mL) incubated at room temperature for different durations (0, 5, 10, 20, 30, and 40 min), as shown in the quantitative results (Fig. 3h). The experimental findings consistently demonstrated that QECQDs exhibit superior ability in clearing ABTS cation and DPPH radicals compared to Que.

### Neuroprotective effects of QECQDs in vitro

**Fig. 4:**
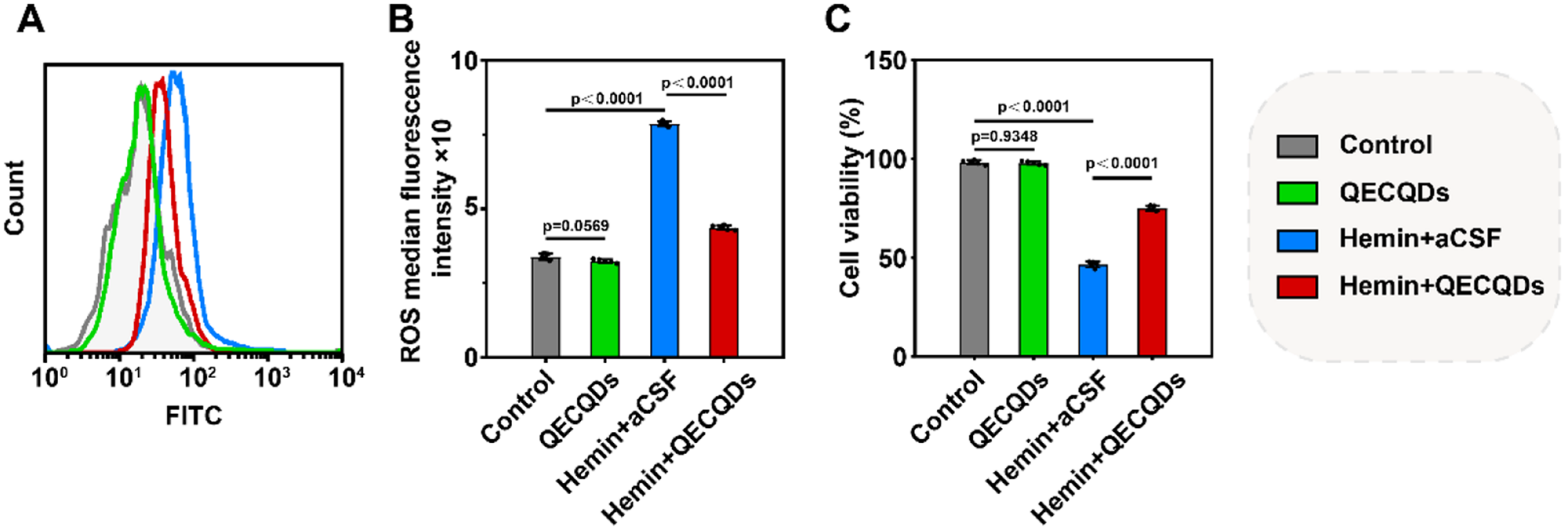
Protective effects of QECQDs on HT22 cells. (**a**) Flow cytometry analysis and (**b**) quantitative analysis of ROS fluorescence intensity in HT22 cells (*n* = 5/group, one-way ANOVA). (**c**) Evaluation of cell viability under different treatments using CCK-8 assay (*n* = 5/group, one-way ANOVA)

Hemin is a lipophilic oxidant known for its strong neurotoxicity [33]. Following ICH, ruptured red blood cells lead to rapid accumulation of hemin within the cranial cavity, triggering oxidative stress and cellular damage, thereby causing injury or death to cells such as neurons and astrocytes [34, 35]. In our research, immortalized HT22 cells derived from the hippocampus were utilized to investigate the neuroprotective effect of QECQDs in clearing intracellular free radicals. HT22 cells are widely employed to study neurotoxicity, oxidative stress, and neuroprotective mechanisms [36–39]. The hemin-treated group exhibited significantly elevated intracellular ROS levels compared to the control group, indicating oxidative stress induced by hemin treatment. Conversely, the hemin + QECQDs-treated group showed markedly reduced intracellular ROS levels compared to the hemin-treated group, demonstrating effective clearance of intracellular ROS by QECQDs (Fig. 4a, b). Additionally, viability assessed by the CCK-8 assay showed that QECQDs treatment significantly attenuated the decrease in neuronal cell viability induced by hemin treatment (Fig. 4c). These findings underscore the potent ROS scavenging capability of QECQDs, reducing hemin-induced neuronal damage and highlighting their neuroprotective efficacy.

### QECQDs distribution post intrathecal injection

**Fig. 5:**
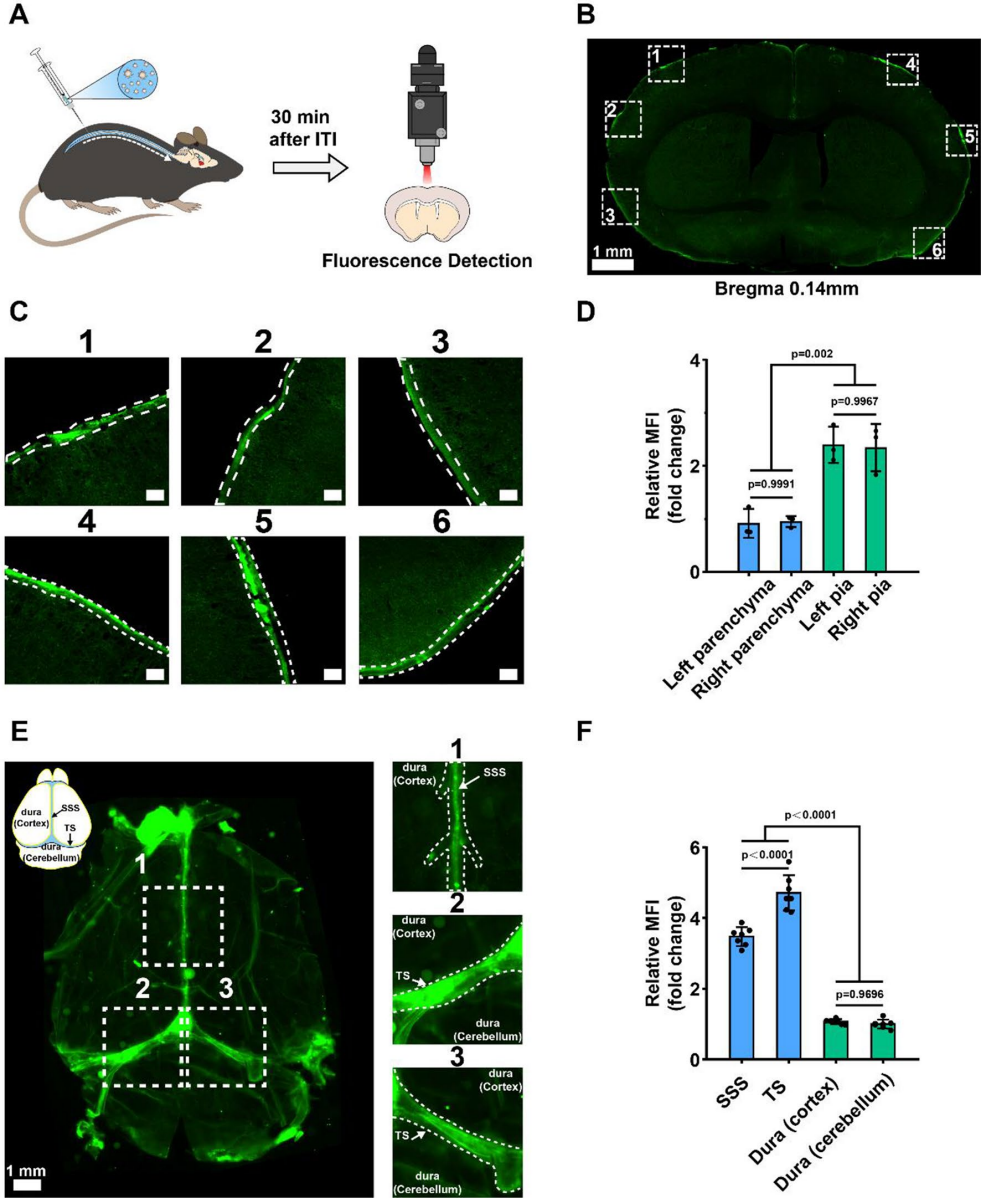
Distribution of QECQDs in the CNS. (**a**) Schematic diagram of the distribution of QECQDs in the CNS 30 min following intrathecal injection. (**b**, **c**) Representative fluorescence images of brain tissue sections after intrathecal injection of QECQDs. Scale bar, 1 mm. Dashed box (**c**) shows an enlarged view of the brain parenchyma edge region. Scale bar, 100 μm. (**d**) Fluorescence quantitative analysis of the brain parenchyma and meningeal system in selected areas on the left and right sides (*n* = 3/group, two-tailed Student’s t-test). (**e**) Representative fluorescence images of dura mater tissue after intrathecal injection of QECQDs. The upper left inset shows the various regions of the dura mater and the superior sagittal and transverse sinuses. The dashed boxes correspond to the magnified fields of different regions of the dura mater in the right image. SSS: superior sagittal sinus, TS: transverse sinus. Scale bar, 100 mm. (**f**) Quantitative fluorescence analysis of the superior sagittal sinus, transverse sinus, dura mater (cortex), and dura mater (cerebellum) (*n* = 7/group, one-way ANOVA)

Intrathecal drug administration involves injecting drugs directly into the central nervous system, circumventing the blood-brain barrier [40, 41]. To investigate the distribution of QECQDs in the CNS, mice were administered QECQDs intrathecally, and brain tissue sections were prepared 30 min post-administration. The distribution of QECQDs in the CNS was monitored using a digital slice scanning imaging system(Fig. 5a). Figure 5b and c show that QECQDs were primarily observed along the brain tissue edges near the pial surface and subarachnoid space, with fluorescence intensity noticeably more potent at the brain tissue edges than at the parenchyma. Quantitative analysis confirmed higher fluorescence intensity along the brain tissue edges compared to the brain parenchyma, demonstrating a predominant accumulation of QECQDs in the pial surface and subarachnoid space (Fig. 5d).

The meningeal system, primarily composed of the dura mater, arachnoid mater, and pia mater, is crucial for maintaining brain homeostasis and function [42]. Therefore, we further investigated the distribution of quantum dots on the dura mater. Through whole-dura mater fluorescence imaging and quantitative analysis, we found a significant accumulation of QECQDs in the superior sagittal sinus and transverse sinus of the dura mater, indicating their presence in the perivascular spaces of the dura (Fig. 5e). These results suggest that QECQD treatment aids in the clearance of iron ions and oxygen free radicals from the perivascular spaces, thereby improving the vascular microenvironment.

### Improvement of brain meningeal blood flow by QECQDs

**Fig. 6:**
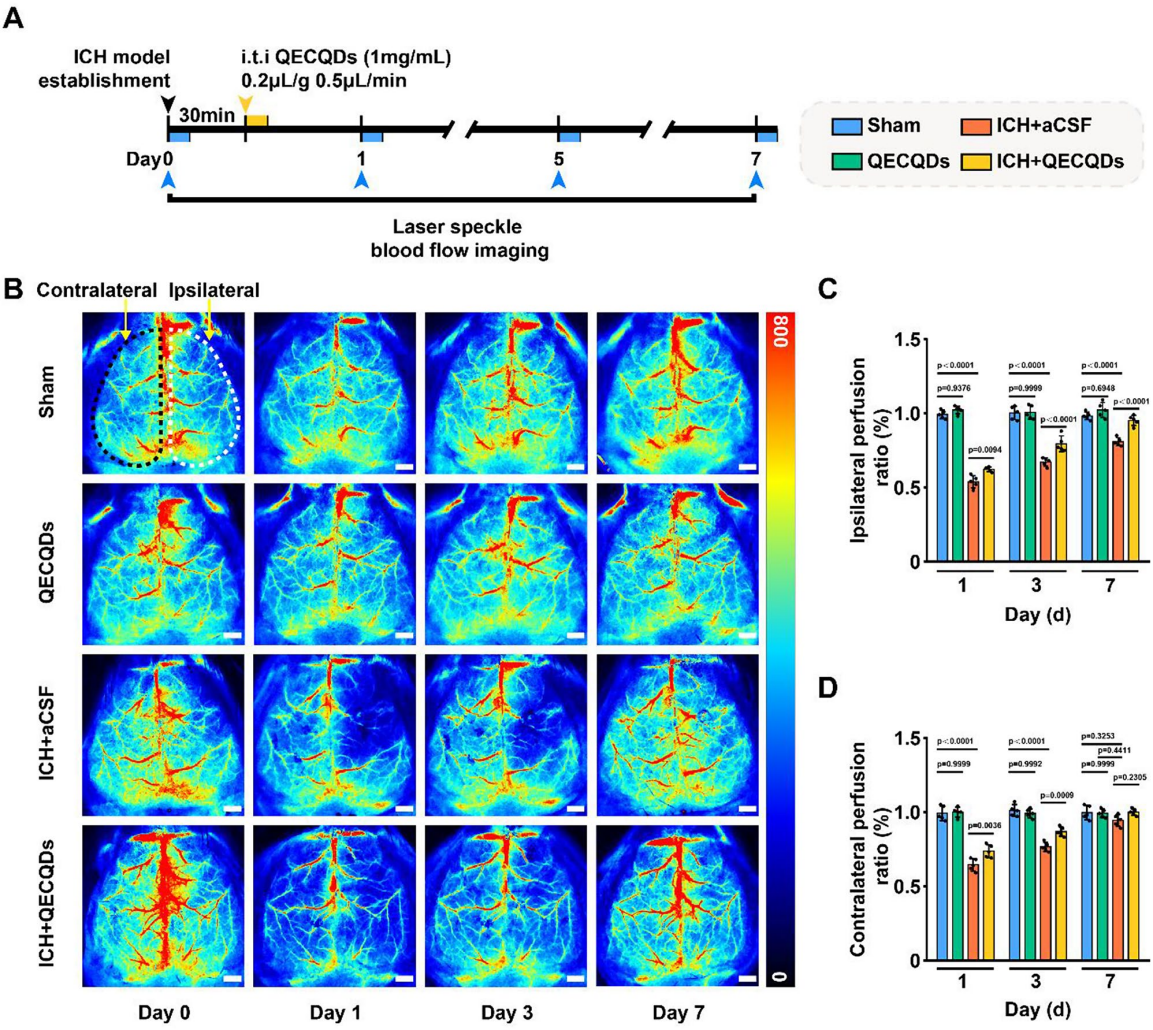
QECQDs promote restoration of meningeal blood perfusion post-ICH. (**a**) Schematic timeline of LSI assessments following intrathecal injection of QECQDs in ICH model. (**b**) Representative LSI images showing meningeal blood perfusion at different time points (day 0, 1, 3, 7). Scale bar,1 mm. (**c**, **d**) Quantitative analysis of meningeal blood perfusion changes over time in the ipsilateral (**c**) and contralateral (**d**) sides (*n* = 5/group, one-way ANOVA)

Laser speckle blood flow imaging system (LSI) is a technique used for real-time monitoring and imaging of tissue blood flow, widely employed in cerebral blood flow studies in mice [43–46]. As shown in Fig. 6a, LSI was performed at different time points before and after ICH to investigate the effect of QECQDs treatment on improving cerebral surface perfusion. LSI detection and quantitative analysis revealed that overall cerebral surface blood flow decreased following hemorrhage induction compared to the sham surgery group. More pronounced reductions in blood flow were observed on the injured side compared to the contralateral side, attributable to increased accumulation of metabolic waste and inflammatory factors in the injured hemisphere [47] (Fig. 6b). On days 1, 3, and 7 post-ICH, compared to the ICH group, the ICH + QECQDs treatment group exhibited significantly increased blood flow in the injured hemisphere (Fig. 6c, d). These data suggest that QECQDs treatment promotes faster recovery of meningeal blood flow after ICH, thereby increasing the discharge of toxic substances, promoting functional recovery of the injured area, and alleviating secondary damage caused by ICH.

### QECQDs improve injury microenvironment

**Fig. 7:**
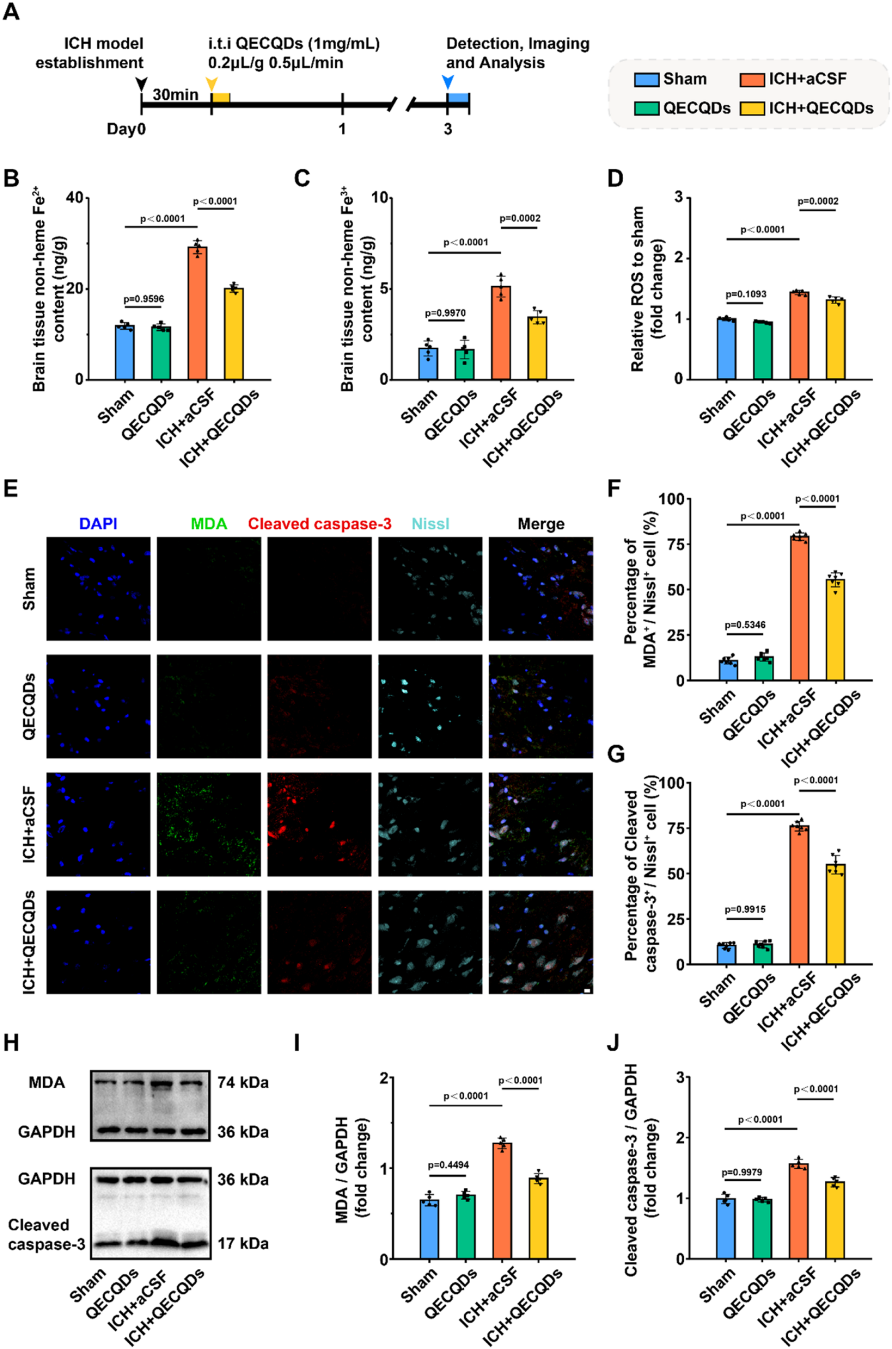
Effects of QECQDs treatment on enhancing the post-ICH microenvironment. (**a**) Schematic timeline of treatment and analysis following ICH model establishment. Inhibition of Fe2^+^ (**b**) and Fe3^+^ (**c**) production induced by ICH on day 3 post-QECQDs treatment (*n* = 5/group, one-way ANOVA). (**d**) Inhibition of ROS production induced by ICH on day 3 post-QECQDs treatment (*n* = 5/group, one-way ANOVA). (**e**) Representative immunofluorescence images illustrating levels of MDA and cleaved caspase-3 in neurons around the hemorrhagic injury site on day 3 post-QECQDs treatment. Neurons are marked by Nissl staining, MDA by FITC, cleaved caspase-3 by TRITC, and nuclei by DAPI. Scale bar, 10 μm. (**f**, **g**) Quantification of MDA^+^ (**f**) and cleaved caspase-3^+^ (**g**) neurons on day 3 post-QECQDs treatment (*n* = 7/group, one-way ANOVA). (**h**) Immunoblot bands and (**i**, **j**) quantitative analysis illustrating levels of MDA (**i**) and cleaved caspase-3 (**j**) in brain tissue 3 days following the QECQDs therapeutic intervention (*n* = 5/group, one-way ANOVA)

After ICH, hemoglobin releases a large amount of iron ions, which catalyze the production of reactive oxygen species (ROS) through the Fenton reaction in vivo. ROS interacts with cellular proteins, DNA, and lipid molecules, leading to oxidative stress damage, a critical factor in inducing neuronal apoptosis and necrosis [3, 4]. As shown in Fig. 7a, we established a mouse model of ICH and administered different treatments to investigate the therapeutic efficacy and mechanisms of QECQDs in treating ICH. Our study revealed a significantly higher accumulation of iron ions (Fe^2+^ and Fe^3+^) in the ICH group compared to the control group. Notably, compared to the ICH group, the ICH + QECQDs treatment group exhibited markedly lower accumulation of iron ions (Fe^2+^ and Fe^3+^) (Fig. 7b, c). These results demonstrate that QECQDs treatment reduces the accumulation of iron ions (Fe^2+^ and Fe^3+^) in brain tissues post-ICH. On the third day after different treatments, the ROS levels in brain tissues of the ICH group and ICH + QECQDs treatment group were 1.43-fold and 1.31-fold higher, respectively, compared to the sham surgery group (Fig. 7d). Compared to the ICH group, the ICH + QECQDs treatment group showed significantly reduced ROS levels in brain tissues, indicating that QECQDs treatment mitigates oxidative stress levels post-bleeding. The above experiments show that QECQDs can effectively remove massive iron ions and ROS in brain tissue in the ICH model and efficiently improve the microenvironment of hemorrhage injury.

To evaluate the neuroprotective effects of QECQDs treatment, we analyzed malondialdehyde (MDA) and Cleaved caspase-3 expression in the brain injury microenvironment(Fig. 7e). This is because lipids can undergo oxidation to generate significant amounts of malondialdehyde (MDA), a crucial marker for assessing the severity of oxidative stress [48, 49]. The Caspase-3 family comprises critical molecules that execute cell apoptosis, with Cleaved caspase-3 being a pivotal marker of activated apoptosis pathways. Unlike the sham group, the proportion of Cleaved caspase-3^+^ and MDA^+^ neurons among Nissl^+^ neurons markedly elevated following ICH. In contrast, the ICH + QECQDs treatment group exhibited significantly fewer Cleaved caspase-3^+^ and MDA^+^ neurons than the ICH group(Figs. 7f, g). These data indicate that QECQDs treatment effectively inhibits oxidative stress-induced neuronal damage and apoptosis. Furthermore, western blot experiments showed that the levels of cleaved caspase-3 and MDA protein expressions were significantly lower in the ICH + QECQDs treatment group than in the ICH group (Fig. 7h-j), suggesting that QECQDs therapy alleviates oxidative stress damage, improves the injury microenvironment post-ICH, reduces neuronal apoptosis, and protects neurons.

### QECQDs treatment alleviates brain injury and improves neurological function

**Fig. 8:**
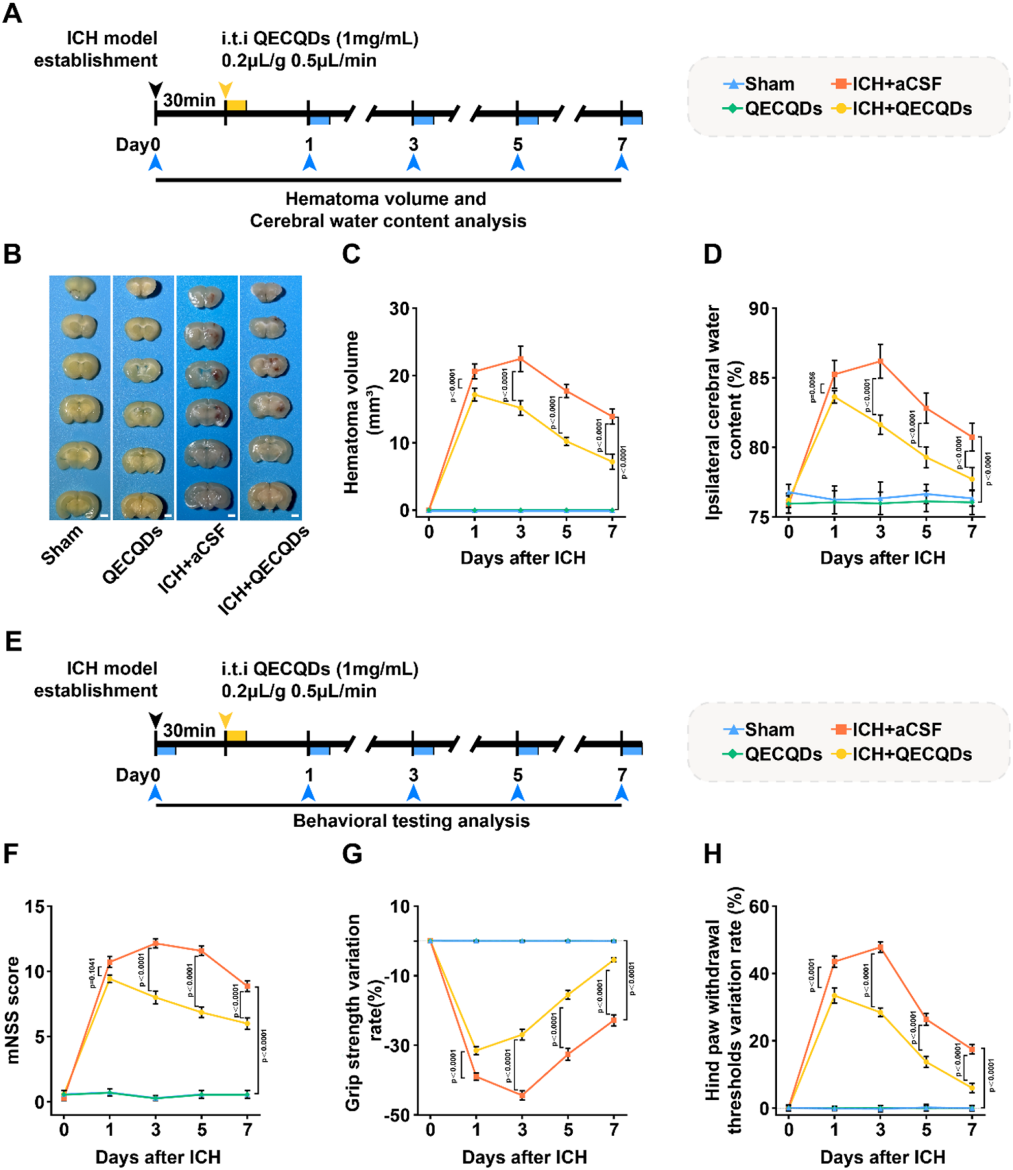
QECQDs treatment alleviates brain injury and enhances neurological function following ICH. (**a**) Schematic diagram of brain injury assessment process following intrathecal injection of QECQDs in the ICH mouse model. (**b**, **c**) Representative images (**b**) and quantitative analysis (**c**) of brain hematoma volume reduction on day 3 post-QECQDs treatment (*n* = 7/group, one-way ANOVA). Scale bar represents 1 mm. (**d**) Quantitative analysis of brain tissue water content changes on days 1, 3, 5, and 7 post-QECQDs treatment in the ipsilateral hemisphere (*n* = 7/group, one-way ANOVA). (**e**) Schematic diagram of neurological function assessment process following intrathecal injection of QECQDs in the ICH mouse model. (**f**) mNSS functional assessment at different time points (days 1, 3, 5, and 7) post-QECQDs intervention (*n* = 7/group, one-way ANOVA). (**g**, **h**) Evaluation of forelimb grip strength (**g**) and left hind limb pain threshold changes (**h**) at different time points (days 1, 3, 5, and 7) post-treatment with QECQDs (*n* = 5/group, one-way ANOVA)

After ICH, the influx of vascular-derived fluids into brain tissue is a significant cause of hematoma and edema [50]. As illustrated in Fig. 8a, we further investigated the therapeutic intervention of QECQDs at different time points post-hemorrhage (days 1, 3, 5, and 7) and its effects on hematoma volume and brain tissue swelling. Our study (Fig. 8b, c) demonstrated that the hematoma volume significantly increased in the ICH group compared to the sham-operated group on days 1, 3, 5, and 7. Significantly, the hematoma volume in the ICH + QECQDs treatment group was reduced compared to the ICH group, showing reductions of 3.44%, 7.30%, 7.47%, and 6.41% on days 1, 3, 5, and 7, respectively. Further investigation revealed (Fig. 8d) that, compared to the sham-operated group, the water content in the ipsilateral brain tissue significantly increased in the ICH group on days 1, 3, 5, and 7. Notably, the water content in the ipsilateral brain tissue of the ICH + QECQDs treatment group was significantly reduced compared to the ICH group, showing reductions of 1.61%, 4.56%, 3.53%, and 2.89% on days 1, 3, 5, and 7, respectively. Additionally, administration of QECQDs alone did not result in significant changes in hematoma volume or ipsilateral brain tissue water content compared to the sham group, indicating good biocompatibility of QECQDs.

To assess the therapeutic effects of QECQDs on neurological function (Fig. 8e), we conducted a modified Neurological Severity Score (mNSS) test [51], forelimb grip strength tests [52], and left hindlimb pain threshold tests [53]. As shown in Fig. 8f, the mNSS score in the ICH group significantly increased (1, 3, 5, and 7 days) post-hemorrhage in comparison to the sham group, revealing severe neurological dysfunction. Interestingly, following QECQDs intervention, the mNSS scores were significantly reduced (1, 3, 5, and 7 days) post-hemorrhage in comparison to the ICH group, suggesting that QECQDs treatment positively influences neurological recovery post-ICH. The forelimb grip strength test, used to assess motor function impairment in mice, showed lower values, indicating more significant damage. Our study revealed that the forelimb grip strength significantly decreased in the ICH group (1, 3, 5, and 7 days) in comparison to the sham-operated group. Conversely, the forelimb grip strength in the ICH + QECQDs group significantly increased (1, 3, 5, and 7 days), with respective average increases of 7.51%, 17.45%, 17.11%, and 17.37% in comparison to the ICH group (Fig. 8g), implying a favorable influence of QECQDs therapy on motor function restoration. The left hindlimb pain threshold test, used to assess sensory system improvement, showed higher values indicating more significant damage. Our study found that the left hindlimb pain threshold increased in the ICH group (1, 3, 5, and 7 days) in comparison to the sham-operated group. Interestingly, the left hindlimb pain threshold in the ICH + QECQDs group significantly decreased (1, 3, 5, and 7 days) in comparison to the ICH group, with respective reductions of 10.08%, 19.37%, 12.65%, and 11.48% (Fig. 8h), indicating that QECQDs treatment significantly improves sensory function impairment.

Finally, we evaluated the biocompatibility of QECQDs. HE staining of mouse organs revealed no significant pathological changes in the QECQDs group compared with the Sham + aCSF group (Fig. S1). Furthermore, Small animal ultrasound results show that intrathecal administration of QECQDs does not elicit significant changes in heart rate (Figure S2b-d), respiratory rate (Figure S2e-S2g), and electrocardiogram readings (Figure S2h) in mice, further corroborating the favourable biocompatibility of QECQDs.

## Conclusion

QECQDs exhibit excellent iron chelation and antioxidant properties, protecting HT22 cells from hemin-induced oxidative stress by reducing ROS accumulation and enhancing cell viability. After intrathecal injection of QECQDs for treating ICH, QECQD treatment not only scavenges excess iron ions and oxygen radicals in the cerebrospinal fluid but also promotes restoration of cerebral surface blood flow, facilitates clearance of metabolic waste and toxic agents in the cerebrospinal fluid. Therefore, QECQD treatment improves the injury microenvironment, protects neurons, alleviates hematoma, and reduces brain tissue edema associated with ICH, thus aiding neurological recovery.

## Materials and methods

### Synthesis of QECQDs

QECQDs were synthesized using a straightforward one-step hydrothermal method [54]. Initially, 0.151 g of quercetin was thoroughly mixed with 10 mL of deionized water and 1 mL of anhydrous ethylenediamine under magnetic stirring. The homogeneous solution was then transferred to a steel autoclave lined with PTFE and heated at 200 °C in an oven for 12 h. After cooling to room temperature, the reaction mixture was centrifuged at 15,000 rpm for 20 min, and the supernatant was collected. The supernatant was subsequently filtered through a water-based microporous membrane filter with a pore size of 0.22 μm. The resulting solution was dialyzed using dialysis bags with a molecular weight cutoff of 500 Da for 48 h, followed by vacuum drying at 60 °C for 24 h. Finally, the concentrate was freeze-dried to obtain brown QECQDs.

### Characterization of QECQDs

The morphology and size of QECQDs under an acceleration voltage of 200 kV were analyzed using a transmission electron microscope (Hitachi HF-3300, Tokyo, Japan). The fluorescence properties of QECQDs were evaluated using a fluorescence spectrophotometer (F-4700, Hitachi) to assess their three-dimensional photoluminescence spectra.

### Hydrodynamic size and zeta potential of QECQDs

The hydrodynamic diameter and zeta potential stability of QECQDs in aqueous suspension were measured using a NanoBrook 90Plus PALS (BROOKHAVEN) instrument. QECQDs were dispersed in deionized water, followed by 20 min of ultrasonication to ensure uniform dispersion. Subsequently, the solution was filtered through a pre-rinsed sterile 0.22 μm filter and equilibrated at room temperature for 10 min before measurement. The aqueous nanoparticle dimensions and zeta potential were assessed at specific time intervals (0, 7, 14, 21, and 28 days post-synthesis) to assess the temporal stability and size characteristics of QECQDs.

### In vitro fluorescence quenching assay of QECQDs with Fe2+/3+

Using a fluorescence spectrophotometer (F-4700, Hitachi), the chelation-induced fluorescence quenching of QECQDs by Fe^2+/3+^ was quantitatively assessed. Specifically, a 1 mg/mL solution of QECQDs was prepared in artificial cerebrospinal fluid. Various concentrations of Fe^2+/3+^ (0, 10, 20, 30, 40, and 50 µM) were added separately, thoroughly mixed, and allowed to react. The emission spectra were then measured under excitation at 400 nm to evaluate the fluorescence spectral changes induced by ion chelation. The observed decline in fluorescence intensity indirectly indicates the iron ion chelation capability exhibited by QECQDs.

### In vitro antioxidant capacity assay of QECQDs

The total antioxidant capacity of quercetin-derived carbon quantum dots (QECQDs) was evaluated using the ABTS assay kit (E2006, APPLYGEN) for liquid samples. Initially, a mixture of ABTS (2,2’-azino-bis (3-ethylbenzothiazoline-6-sulfonic acid)) solution and oxidant was prepared in equal volumes under dark conditions and allowed to incubate for 6 h to generate the ABTS+· working solution. Subsequently, varying concentrations of QECQDs/Quercetin solutions (0, 1, 2.5, 5, 10, and 20 µg/mL) were thoroughly mixed with the ABTS+· working solution and incubated for 15 min. The optical density was assessed at 734 nm using a PerkinElmer Victor Nivo multimode microplate reader (PerkinElmer, Waltham, MA), with absorbance values (A0) measured using phosphate-buffered saline (PBS) as the blank control. Additionally, the 20 µg/mL QECQDs/Quercetin solution was mixed with the ABTS+· working solution and incubated for different durations (0, 5, 10, 15, 20, and 25 min) to assess the time-dependent antioxidant capacity of QECQDs. The percentage of ABTS+· clearance rate was calculated using the formula: (A1 - A0) / A1 × 100%, where A1 represents the absorbance of the sample and A0 represents the absorbance of the blank control.

To evaluate the free radical scavenging capabilities of quercetin and QECQDs, the DPPH radical scavenging assay kit (AKAO020M, Boxbio) was employed. Initially, the DPPH working solution was prepared according to the instructions in the kit. Subsequently, different concentrations of QECQDs/Quercetin solutions (0, 1, 2.5, 5, 10, and 20 µg/mL) were thoroughly mixed with the DPPH· working solution and incubated at room temperature for 30 min. The absorbance was measured at 515 nm using a multimode microplate reader, with absorbance values (AII) measured using PBS as the blank control (AI). Additionally, the 20 µg/mL QECQDs/Quercetin solution was mixed with the DPPH· working solution and incubated for different durations (0, 5, 10, 20, 30, and 40 min) to assess the time-dependent antioxidant capacity of QECQDs. The DPPH· clearance rate percentage was calculated using the formula: (AII - AI) / AII × 100%.

### HT22 cell culture preparation

HT22 cells were cultured in Dulbecco’s Modified Eagle Medium (DMEM) supplemented with 10% fetal bovine serum (FBS), 100 U/mL penicillin, and 100 µg/mL streptomycin. Cells were maintained at 37 °C in a humidified atmosphere containing 5% CO_2_.

### Construction and grouping of HT22 cell injury model

HT22 cells were subjected to various concentrations (0, 10, 20, 40, 60, 80, 100, and 120 µM) of Hemin stimulation for 24 h to establish a neuronal injury model. Cell viability was evaluated using the CCK-8 assay, revealing cell viability close to 50% following treatment with 100 µM Hemin. Subsequently, an in vitro cell injury model resembling intracerebral hemorrhage (ICH) was established using 100 µM Hemin, and the cells were divided into four groups as follows: (1) Control group: 0 µg/mL QECQDs + 0 µM Hemin; (2)QECQDs group: 20 µg/mL QECQDs + 0 µM Hemin; (3)Hemin + aCSF group: 0 µg/mL QECQDs + 100 µM Hemin + an equal volume of aCSF; (4)Hemin + QECQDs group: 20 µg/mL QECQDs + 100 µM Hemin. All cell groups were cultured in DMEM for 36 h, and cell viability was assessed using the CCK-8 assay. Subsequently, intracellular reactive oxygen species (ROS) levels were measured using a 10 µM DCFH-DA fluorescent probe and quantified utilizing flow cytometry.

### Distribution of QECQDs in the CNS

To explore the distribution pattern of QECQDs in the brain, intrathecal injection of QECQDs was administered at a dosage of 200 ng/g (based on the body weight of the mice). Thirty minutes following the injection, mice were transcardially perfused with PBS at room temperature, followed by perfusion with pre-chilled PBS containing 2.5% paraformaldehyde. Immediately after perfusion, the skull was carefully removed, and brain tissue was collected. The harvested tissue was then fixed in 4% paraformaldehyde for 24 h and subsequently sectioned using a cryostat (CM1950, Leica). The brain sections were further examined using a digital slide scanning system (VS 120, Olympus) under an excitation wavelength of 365 nm to visualize the distribution of QECQDs.

### Distribution of QECQDs in the dura mater

To study the distribution of QECQDs in the meninges, QECQDs were injected intrathecally at a dose of 200 ng/g (based on the body weight of the mice). After 30 min of injection, the mice were perfused transcardially with pre-cooled PBS, followed by perfusion with pre-cooled PBS containing 2.5% paraformaldehyde. The dermal layer enveloping the mouse cranial vault is excised, and the optic nerve is severed to isolate the eyes. Subsequently, the head is inverted, orienting the ventral aspect upward. Employing scissors, the robust muscles connecting the mandible to the skull via the oral cavity are incised and excised. With curved scissors, the infraorbital foramina on both sides of the skull is meticulously removed, ensuring thorough elimination of all soft tissue adhering to the cranial bones. With utmost care, curved scissors are utilized to execute a counterclockwise incision below the tympanic membrane hook, delicately separating the inferior segment of the skull while maintaining an outward bevel to safeguard the integrity of the brain parenchyma. The nasal bone is subsequently transected anterior to the olfactory bulb. The encephalon is extracted from the superior cranium and immersed in a 4% paraformaldehyde solution for fixation at 4 °C in the dark overnight [55]. After overnight fixation, the skull was transferred to pre-cooled PBS, and the meninges were carefully and completely peeled off, the residual tissue was trimmed and removed, and then washed with PBS. The meningeal tissue was placed on a slide and flattened. Finally, a digital slice scanning system (VS 120, Olympus) was used for observation at an excitation wavelength of 365 nm.

### C57 BL/6 J mouse preparation

All C57 BL/6 J mice were procured from Nanjing Keruisi Animal Co., Ltd. and acclimated under standardized conditions, maintaining a controlled environment with a temperature of 20 ± 2 °C and relative humidity of 50 ± 10%. The mice were provided ad libitum access to both food and water. This study strictly adhered to ethical considerations, following the laboratory animal care and handling guidelines set forth by the National Institutes of Health (NIH). The experimental protocols received approval from the Laboratory Animal Ethics Committee of Nanchang University, with the assigned approval number being NCULAE-20,230,610,001.

### Establishment of C57 BL/6 J mouse ICH model and experimental grouping

For the study, male C57 BL/6 J mice aged 7–9 weeks were utilized as experimental subjects. Anesthesia was initiated with 2.3% isoflurane and subsequently maintained at a concentration of 1.3% isoflurane. The mice were securely positioned in a stereotaxic frame (RWD Life Science Co, Shenzhen, China). A microsyringe was employed to administer an injection of type IV collagenase (67 U/ml, Sigma-Aldrich) into the striatum region of the brain. The injection volume was calculated at 0.04 µl per gram of body weight. The precise coordinates for injection were as follows: 0.38 mm anterior to bregma, 2.0 mm lateral to midline, and a depth of 3.0 mm. The injection was carefully conducted at a controlled rate of 0.5 µL/min [56]. To maintain optimal body temperature throughout the surgical procedure, a heating pad was employed to sustain a temperature of 37 °C in mice. Following the recovery from anesthesia, the mice were closely monitored for a specified period before being returned to their respective housing conditions in compliance with appropriate animal care guidelines.

### Randomization and grouping of mice

The mice were subjected to random allocation into four distinct groups based on the experimental design: (1) Sham surgery group: In this group, the mice received an intrastriatal injection of 5 µl of sterile saline solution, precisely administered at the coordinates of 0.38 mm anterior to bregma, 2.0 mm lateral to midline, and a depth of 3.0 mm, delivered at a controlled rate of 0.5 µL/min. Thirty minutes following the initial injection, an additional 5 µl of aCSF was intrathecally administered. (2) QECQDs group: Similar to the sham surgery group, the mice underwent an intrastriatal injection of 5 µl of sterile saline solution at the coordinates above and rate. After a 30-minute interval, the mice received an intrathecal injection of QECQDs at a concentration of 1 mg/ml, with a dose of 0.2 µL/g based on the injection volume per mouse weight. (3) ICH + aCSF group: the mice underwent intrastriatal injection of collagenase using the same coordinates and rate as previously described. Thirty minutes following the collagenase injection, a total volume of 5 µl of aCSF was intrathecally administered. (4) ICH + QECQDs group: Similar to the ICH + aCSF group, the mice received intrastriatal collagenase injection at the specified coordinates and rate. After 30 min, QECQDs at a concentration of 1 mg/ml were intrathecally injected at a dose of 0.2 µL/g, taking into account the injection volume relative to the mouse weight.

### Evaluation of cerebral surface blood flow in C57 BL/6 J mice

Real-time visualization of brain surface blood flow in mice was performed using a laser speckle blood flow imaging system (RFLSI III, Shenzhen RWD Life Sciences Co., Ltd.). Briefly, mice underwent scalp incision under 1.3% isoflurane anesthesia to expose the cranial bones from bregma to lambdoid, the surface was evenly coated with coupling agent, and residual hair was carefully removed. A 785 nm monitoring laser was focused on the skull to assess mean blood perfusion over 30 s.

### Detection of brain tissue iron ion content

On the 3rd day following the induction of the ICH model, brain tissue samples were meticulously collected for analysis. The quantification of divalent/trivalent iron ions in the tissue was performed using the total iron ion colourimetric assay kit (Elabscience, E-BC-K772-M) and the ferrous iron ion colourimetric assay kit (Elabscience, E-BC-K773-M), following the specific instructions provided by the manufacturer. The concentration of ferric iron ions was subsequently calculated by subtracting the ferrous iron ion content from the total iron ion content, allowing for a comprehensive assessment of iron ion homeostasis in the brain tissue.

### Detection of reactive oxygen species (ROS) in brain tissue

A ROS detection assay kit (C1300, APPLYGEN) was employed to quantify ROS levels in brain tissue. On day 3 post-treatment, brain tissues were harvested and immediately homogenized in pre-chilled PBS at a ratio of 1:9 (w/v). Single-cell suspensions were rapidly prepared at 4 °C, washed three times with PBS, and centrifuged to collect the cell pellets. The pellets were resuspended in PBS, and a 10 µM DCFH-DA probe was added to the cell suspension [57]. The samples were then incubated at 37 °C for 1 h with thorough mixing every 10 min. After centrifugation at 1000 g for 5 min, the supernatant was discarded, and the cell pellets were washed three times with PBS and resuspended. ROS levels in brain tissue cells were measured using a 500 nm excitation wavelength.

### Immunofluorescence staining

Following the intervention, mouse brain tissues were meticulously prepared, fixed in 4% paraformaldehyde for 48 h, and subsequently processed into 20 μm sections using a cryostat (CM1950, Leica). The sections were washed with PBS, incubated in a blocking solution at room temperature for 2 h, and then treated overnight at 4 °C with primary antibodies against cleaved caspase-3 and anti-MDA while avoiding exposure to light. After washing with PBS, the sections were incubated with secondary antibodies conjugated with Fluorescein isothiocyanate (FITC) and Tetramethylrhodamine isothiocyanate (TRITC) for 2 h at room temperature in the dark. Following PBS washes, the sections were stained for neurons with DAPI and Nissl. Quantification of MDA^+^ and cleaved caspase-3^+^ neurons was performed using Fiji ImageJ software.

### Western blotting

On the third day post-intervention, lesioned brain tissues were harvested and thoroughly washed with ice-cold PBS to remove blood and contaminants. Tissues were then homogenized on ice and lysed in protein lysis buffer, followed by sonication for 5 min at 4 °C. The lysates were centrifuged, and the supernatants were collected. Protein concentrations were determined using a BCA protein assay kit. Subsequently, samples were denatured by heating at 100 °C for 5 min in an appropriate buffer. The denatured proteins were separated by sodium dodecyl sulfate-polyacrylamide gel electrophoresis (SDS-PAGE) and transferred onto PVDF membranes. Non-specific binding sites on the membranes were blocked with 5% skim milk. The membranes were then incubated with primary antibodies against MDA (ab27642), Cleaved caspase-3 (ab214430), and GAPDH (ab9485). Following incubation with secondary antibodies, protein bands were visualized using the Tanon 5200 enhanced chemiluminescence system.

### Quantification of ICH volume and brain water content

Following the establishment of the ICH model on days 1, 3, 5, and 7 post-ICH induction, the mice were humanely euthanized by cervical dislocation. The brains were meticulously extracted and immersed in 4% paraformaldehyde for 24 h to ensure proper fixation. Subsequently, the brains were coronally sectioned into 1 mm slices utilizing a vibratome instrument (VT 1200 S, LEICA) for precise and consistent slicing. The size of the hemorrhage was determined by measuring the hemorrhage areas using Fiji ImageJ software, and the slice thickness was taken into account to calculate the hematoma volume using the formula: Hematoma volume = sum of hemorrhage areas × slice thickness [58].

To assess the brain tissue water content surrounding the hemorrhage, the wet-dry method was used by us. The wet weight of the damaged cerebral hemisphere was recorded on days 1, 3, 5, and 7 after ICH induction. Subsequently, the tissue samples were dried at 60 °C for 72 h to obtain the dry weight. The brain tissue water content was calculated using the formula: [(wet weight - dry weight) / wet weight] × 100% [59].

### Evaluation of mouse neurological function

At 1, 3, 5, and 7 days following the induction of ICH, the assessment of neurological deficits was conducted employing the modified Neurological Severity Score (mNSS) [60]. This comprehensive scoring system serves as a robust tool to evaluate diverse aspects of neurological function, encompassing motor function, sensory function (tactile, visual, and proprioceptive), balance, and reflexes (such as the corneal reflex, startle reflex, and ear reflex). The scoring system ranges from 0 to 18, with severity stratified into three categories: mild (0–6 points), moderate (7–12 points), and severe (13–18 points).

### Experiment for Hind paw mechanical pain threshold measurement

The hind paw mechanical pain threshold of mice was evaluated using a plantar test system (SA502, Jiangsu Science Biological Technology Co. Ltd.). Mice were placed in the measurement cage for 30 min to acclimate to the environment and reduce stress and interference. A fine needle was vertically placed on the plantar surface of the left hind paw to establish contact. Subsequently, the needle was incrementally elevated until the mouse withdrew its left hind paw in response. The maximum force applied by the needle on the mouse’s hind paw at withdrawal was recorded as the mechanical pain threshold.

### Mouse grip strength assessment

Grip strength testing was employed to evaluate the recovery of neuromuscular function in mice. Briefly, mice were placed on a measurement platform and encouraged to grasp tightly onto a grip strength meter equipped with a force sensor. The peak force was generated by the mouse as it slowly and steadily pulled by its tail until release was recorded.

### Hematoxylin and eosin (HE) staining

To evaluate the biocompatibility of QECQDs, mice were subjected to intrathecal injection of 200 ng/g QECQDs as a pretreatment. After three days, the mice were dissected, and the hearts, livers, spleens, kidneys, and lungs were collected. The collected organs were fixed in paraformaldehyde for 24 h and dehydrated in a gradient series of ethanol solutions (70%, 80%, 90%, 95%, 100%, 100%). Subsequently, 10 μm thick tissue sections were obtained using a cryostat (CM1950, Leica) and rehydrated in a reverse gradient series of ethanol solutions (100%, 100%, 95%, 90%, 80%, 70%). The sections were stained with hematoxylin for 4 min, followed by double-distilled water rinses for 4 min. Next, differentiation was achieved by treating the sections with 1% hydrochloric acid-alcohol differentiation solution for 3 s, followed by immersion in a 1% ammonium hydroxide solution for 1 min for counterstaining. The sections were then stained with eosin for 1 min and dehydrated in a gradient series of ethanol solutions (70%, 80%, 90%, 95%, 100%, 100%). Finally, the tissue sections were mounted in a neutral mounting medium and observed under an optical microscope (DMi8, Leica).

### Evaluating the physiological parameters of mice

The treatment groups received intrathecal injections of QECQDs at doses of 100 ng/g and 200 ng/g (dose/body weight) respectively, while the Sham group received an equivalent volume of aCSF. Using the Vevo 3100LT ultrasound system (VisualSonics Inc., Toronto, Canada), the mice were monitored for changes in heart rate, respiratory rate, and electrocardiogram (ECG) patterns under different leads, with measurements taken every day from day 0 to day 5.

### Statistical analysis

Data analysis and visualization were performed using Prism 8.0. Statistical significance was determined using a two-tailed Student’s t-test (for two groups) or one-way analysis of variance (ANOVA) (for three or more groups). A p-value < 0.05 was considered statistically significant. Asterisks denote significance levels (*P < 0.05, **P < 0.01, ***P < 0.001, ****P < 0.0001). Data are presented as mean ± standard deviation (SD).

## Electronic supplementary material

Below is the link to the electronic supplementary material.

Supplementary Material 1

Supplementary Material 2

## Data Availability

The data supporting this study will be available upon request.
