# Supplementary material for: Quercetin carbon quantum dots: dual-target therapy for intracerebral hemorrhage in mice
Source: Mol Brain. 2025 Mar 3;18:17. doi: 10.1186/s13041-024-01159-6 (PMC11874443; doi:10.1186/s13041-024-01159-6)
Supplement: Supplementary file 1 — Supplementary Material 1 [file 13041_2024_1159_MOESM1_ESM.docx]

**Quercetin carbon quantum dots: dual-target therapy for intracerebral hemorrhage in mice**

Guangyu Jia1,2,3,4,5***†***, Xinyu Yang2,3,4,5***†***, Yamei Yu1,3,4,5***†***, Yuanyuan Li3,4,5, Zhe Zhang2,3,4,5, Xiaolong Tang 2,3,4,5, Qi Wang2,3,4,5, Heqing Zheng1,3,4,5, Shiyong Li2,3,4,5*, Ye Wang1,3,4,5*

^1^*Department of Neurology, The Second Aﬀiliated Hospital, Jiangxi Medical College, Nanchang University, Nanchang, Jiangxi 330006, China.

^2^Department of Neurosurgery, The Second Aﬀiliated Hospital, Jiangxi Medical College, Nanchang University, Nanchang, Jiangxi 330006, China.

^3^Institute of Neuroscience, Nanchang University, Nanchang, Jiangxi 330006, China.

^4^Jiangxi Province Key Laboratory of Neurological Diseases, Nanchang, Jiangxi 330006, China.

^5^JXHC Key Laboratory of Neurological Medicine, Nanchang, Jiangxi 330006, China.

*Corresponding author(s). E-mail(s): ndefy08041@ncu.edu.cn; [sylicg@ncu.edu.cn](mailto:sylicg@ncu.edu.cn);

Contributing authors: 403007220005@email.ncu.edu.cn; 353007210006@email.ncu.edu.cn; 403007220020@email.ncu.edu.cn; [ndefy21215@ncu.edu.cn](mailto:ndefy21215@ncu.edu.cn); [363007220042@email.ncu.edu.cn](mailto:363007220042@email.ncu.edu.cn); 413007210224@email.ncu.edu.cn; [403007230003@email.ncu.edu.cn](mailto:403007230003@email.ncu.edu.cn); 363007220052@email.ncu.edu.cn;

***†***These authors contributed equally to this work.

**Supplementary materials：**


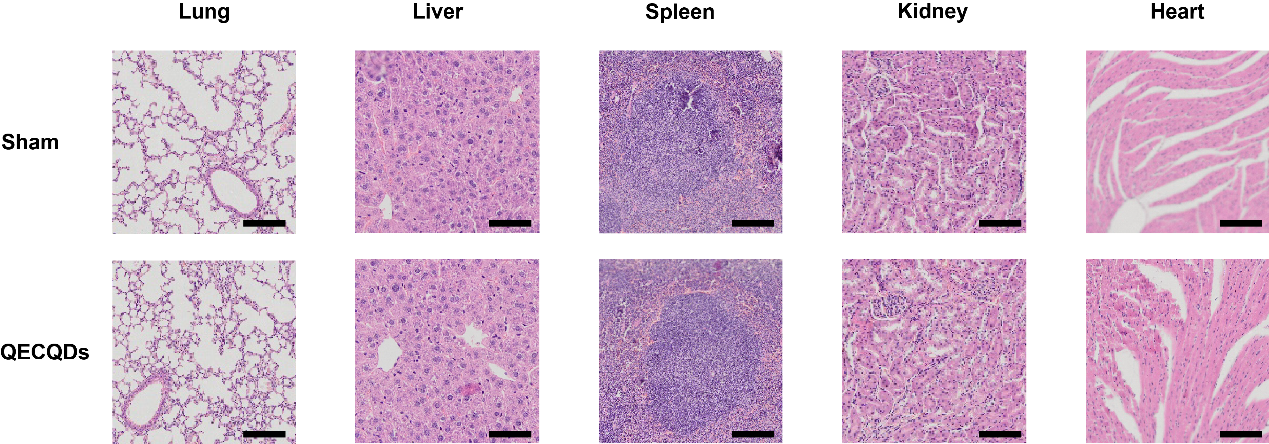


**Fig S1 QECQDs exhibit good biosafety.** H&E-stained tissues of the heart, liver, spleen, lung, and kidney from sham and QECQD-treated mice. Scale bar: 50 μm.


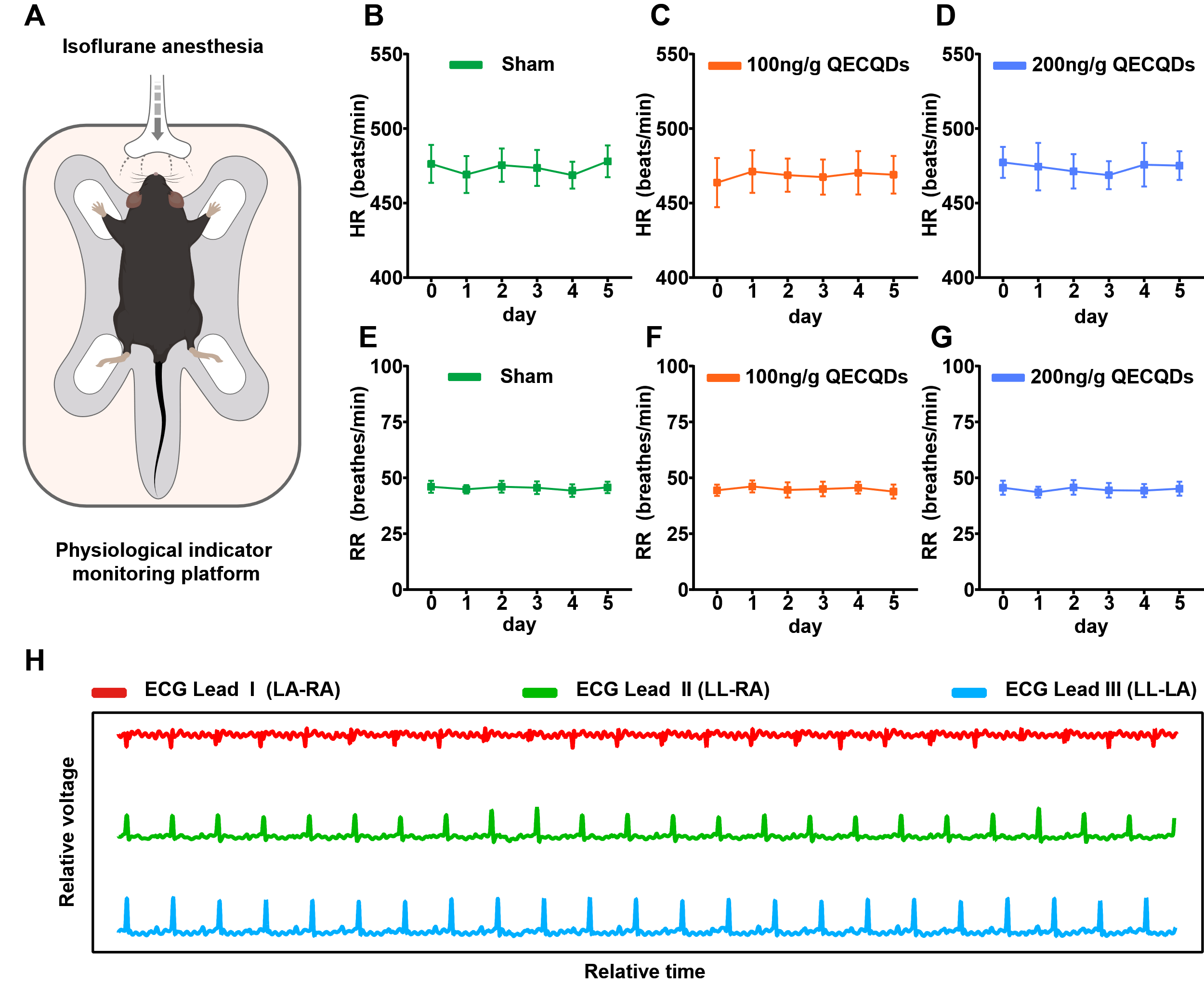


**Fig S2 QECQDs treatment did not affect normal physiological parameters in mice.**

(a) Schematic diagram of electrocardiogram monitoring in mice following intrathecal injection of QECQDs using the Vevo 3100LT ultrasound system. (b-d) Changes in heart rate of mice on days 0, 1, 2, 3, 4, and 5 after intrathecal injection of aCSF (b), 100 ng/g QECQDs (c), and 200 ng/g QECQDs (d) (n = 7/group, one-way ANOVA). (e-g) Changes in respiratory rate of mice on days 0, 1, 2, 3, 4, and 5 after intrathecal injection of aCSF (e), 100 ng/g QECQDs (f), and 200 ng/g QECQDs (g) (n = 7/group, one-way ANOVA). (h) Evaluation of changes in mouse electrocardiograms under different leads following intrathecal injection of QECQDs.


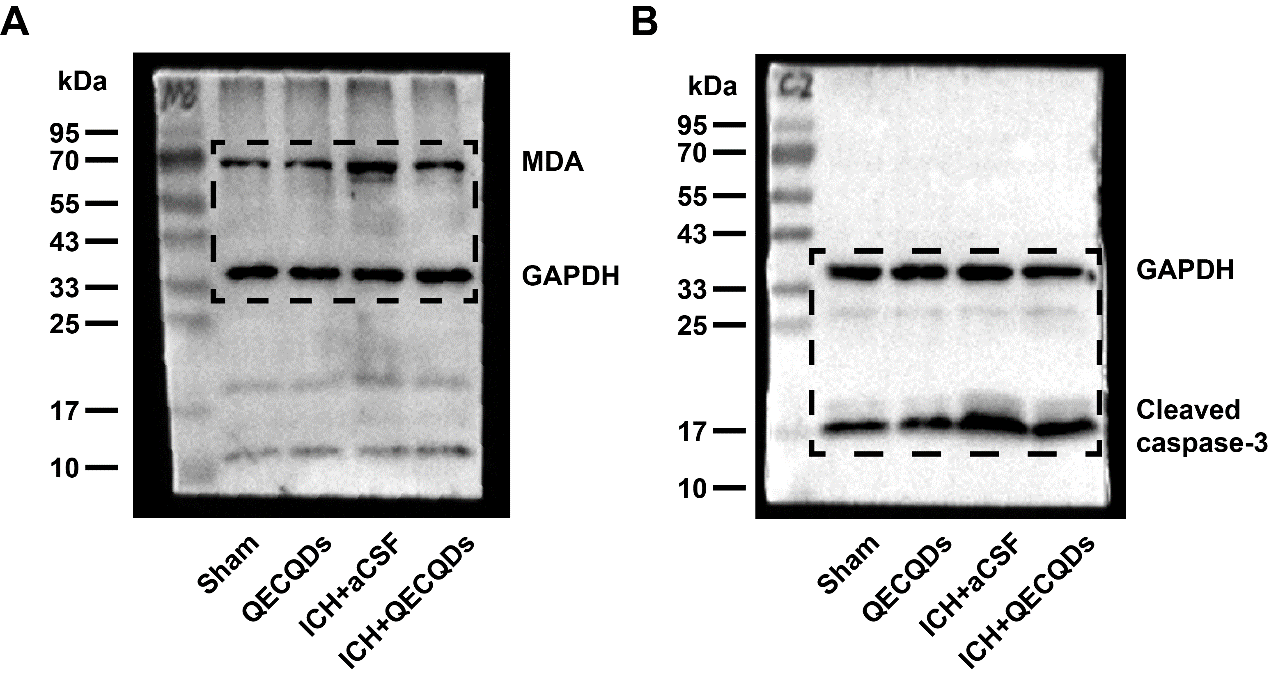


**Fig S3 Immunoblot bands of MDA (A) and Cleaved caspase-3 (B) in brain tissue 3 days following the QECQDs therapeutic intervention.** Western blot images of the selected portion are displayed in Fig 7H.
